# The prevalence of submicroscopic *Plasmodium falciparum* gametocyte carriage and multiplicity of infection in children, pregnant women and adults in a low malaria transmission area in Southern Ghana

**DOI:** 10.1186/s12936-018-2479-y

**Published:** 2018-09-17

**Authors:** Helena Lamptey, Michael Fokuo Ofori, Kwadwo Asamoah Kusi, Bright Adu, Eunice Owusu-Yeboa, Eric Kyei-Baafour, Andrea Twumwaa Arku, Samuel Bosomprah, Michael Alifrangis, Isabella A. Quakyi

**Affiliations:** 1 Immunology Department, Noguchi Memorial Institute for Medical Research, College of Health Sciences, University of Ghana, Legon, Ghana; 2 Department of Biostatistics, School of Public Health, College of Health Sciences, University of Ghana, Legon, Ghana; 3 Centre for Medical Parasitology, Department of Immunology and Microbiology, University of Copenhagen, Copenhagen, Denmark; 4 Department of Infectious Disease, National University Hospital (Rigshospitalet), Copenhagen, Denmark; 5 Department of Biological, Environmental and Occupational Health Sciences, School of Public Health, College of Health Sciences, University of Ghana, Legon, Ghana

**Keywords:** *Plasmodium falciparum*, Gametocyte prevalence, Submicroscopic infections, Multiplicity of infection, Seroprevalence, *Pf*s230, Ghana

## Abstract

**Background:**

The gametocyte stage of *Plasmodium falciparum* is considered an important target for disrupting malaria transmission. Indications are that various demographic groups, such as children and pregnant women may differ in risk of harbouring gametocytes, which may be crucial for targeted control. In this study, the relationship between the prevalence and multiplicity of *P. falciparum*, asexual parasite infections and gametocytaemia was assessed in three different demographic groups in an area of southern Ghana with low malaria endemicity. Levels of antibody responses to *Pf*s230 were also assessed as a proxy for the presence of gametocytes.

**Methods:**

The study involved multiple cross-sectional sampling of children (N = 184, aged 2–15 years), male and non-pregnant female adults (N = 154, aged 16–65 years) and pregnant women (N = 125, aged 18–45 years) from Asutsuare in the Shai Osudoku District of Greater Accra Region in Ghana. Asexual parasitaemia was detected by microscopy and PCR, and gametocytaemia was assessed by *Pf*s25-real time PCR. Multiclonal *P. falciparum* infections were estimated by *msp2* genotyping and an indirect ELISA was used to measure plasma IgG antibodies to *Pf*s230 antigen.

**Results:**

Overall, children and pregnant women had higher prevalence of submicroscopic gametocytes (39.5% and 29.7%, respectively) compared to adults (17.4%). Multiplicity of infection observed amongst children (3.1) and pregnant women (3.9) were found to be significantly higher (P = 0.006) compared with adults (2.7). Risk of gametocyte carriage was higher in individuals infected with *P. falciparum* having both *Pf*msp2 3D7 and FC27 parasite types (OR = 5.92, 95% CI 1.56–22.54, P = 0.009) compared with those infected with only 3D7 or FC27 parasite types. In agreement with the parasite prevalence data, anti-*Pfs*230 antibody levels were lower in gametocyte positive adults (β = − 0.57, 95% CI − 0.81, − 0.34, P < 0.001) compared to children.

**Conclusions:**

These findings suggest that children and pregnant women are particularly important as *P. falciparum* submicroscopic gametocyte reservoirs and represent important focus groups for control interventions. The number of clones increased in individuals carrying gametocytes compared to those who did not carry gametocytes. The higher anti-gametocyte antibody levels in children suggests recent exposure and may be a marker of gametocyte carriage.

**Electronic supplementary material:**

The online version of this article (10.1186/s12936-018-2479-y) contains supplementary material, which is available to authorized users.

## Background

Recent reports on malaria have indicated a 41% decline in incidence rate in sub-Saharan Africa, and a 37% decline globally between 2000 and 2015 [1]. However, malaria remains a major public health concern as it affects children below 5 years and pregnant women, resulting in high morbidity and mortality in these vulnerable groups. Malaria transmission rates vary widely in Ghana, with some areas having low transmission while other areas are hotspots of intense transmission [2–4].

*Plasmodium falciparum* gametocytes develop through five stages (I–V) with different morphological features compared to the asexual parasites. The early stage gametocytes (I–IV) usually sequester in the bone marrow, spleen and other organs to avoid immune clearance [5–7]. The matured stage V gametocyte-infected erythrocyte, which comes back into peripheral circulation, is either picked up by a mosquito for transmission or is degraded in the host.

With the exception of primaquine, most of the currently available anti-malarial drugs target the disease-causing asexual stage parasites and have very limited effect on the liver stage parasites and gametocytes. Studies have shown that *P. falciparum* gametocytes may persist at submicroscopic levels even after treatment with artemisinin-based combination therapy (ACT) and may be effectively transmitted [8, 9]. Effective clearance of gametocytes is necessary for interrupting malaria transmission, and identification of individuals in a population who most likely harbour high gametocyte densities may contribute to malaria elimination.

Children in malaria endemic areas have been shown to have generally higher parasite densities compared to adults [10–12], and younger children, especially in high transmission areas, have higher prevalence of gametocytes compared to older children [13, 14]. Children may therefore, be more efficient gametocyte reservoirs and hence contribute significantly to malaria transmission.

Malaria infection during pregnancy has been demonstrated to result in asexual parasite sequestration in the placenta as an immune evasion strategy [15, 16]. Parasites may accumulate to high densities and form gametocytes, hence pregnant women may form a reservoir of gametocytes for transmission. These collectively indicate a need to assess gametocyte prevalence in endemic communities and to determine which demographic groups are most important gametocyte carriers for the purposes of targeted control.

Another important determinant of gametocyte carriage is infection with multiple clones of *P. falciparum* parasites. The presence of multiple asexual parasite clones may enhance the chance of some clones to evade the immune response, persist in the host and promote gametocyte development [17, 18]. Degradation of the terminal stage V gametocytes exposes gametocyte antigens to the host immune system, with the consequent induction of gametocyte antigen-specific antibody responses. These antibodies would however not be effective against live gametocytes in the host due to their enclosure within the RBC environment, but may rather block parasite development in the mosquito when picked up as part of a blood meal [6, 19]. Such antibodies are expected to be markers for prior exposure to gametocytes [20, 21]. A number of *P. falciparum* sexual stage antigens have been identified and specific antibodies to these have been detected in host plasma [20–22]. *Pf*s230, expressed on matured gametocytes, is one of the leading transmission reducing vaccine candidate antigens and natural anti-*Pfs*230 antibody responses have been demonstrated [7, 21]. These antibodies may provide useful information about the gametocyte exposure history in *Plasmodium* infected individuals.

This study used sensitive molecular tools to investigate the association between asexual parasites and gametocyte prevalence and multiclonal infections among children, adults and pregnant women living in a low malaria endemic area. The study further investigated the levels of anti-*Pf*s230 antibodies as a marker of previous exposure to gametocytes in the three study groups. Since these factors most likely influence gametocyte carriage, which impacts on transmission, it is important to know which demographic group(s) serves as infectious reservoirs for malaria transmission in this low transmission area.

## Methods

### Study area, design and participants

**Fig. 1:**
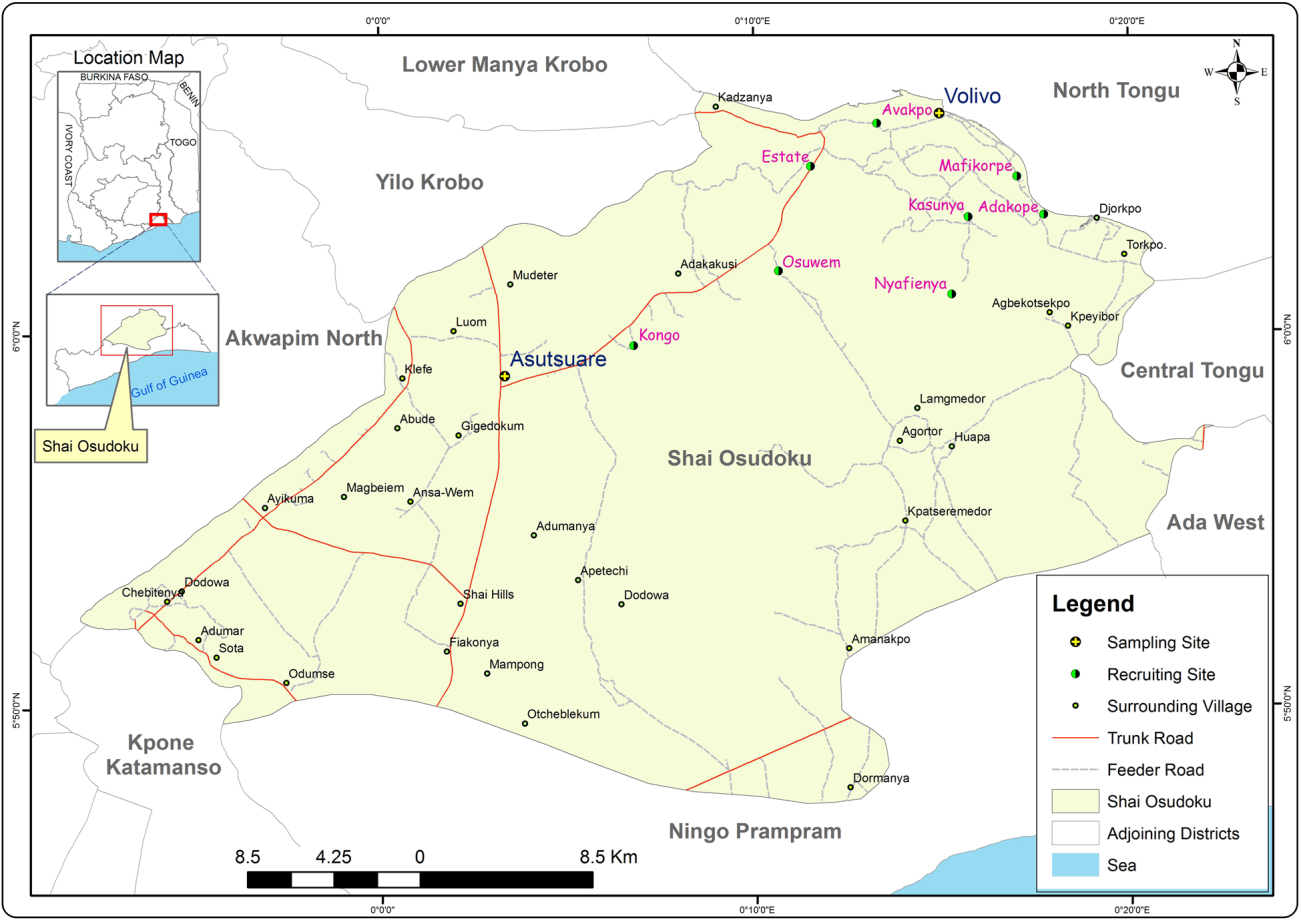
Site Map of Asutsuare in the Shai Osudoku District of Greater Accra Region, Ghana, showing the study sites

The study was conducted in Asutsuare in the Shai Osudoku District of the Greater Accra Region of Ghana. The study area and its surrounding villages including Mafi Korpe, Avakpo, and Volivo, described in details elsewhere (2), are about 120 km north-east of the national capital, Accra (Fig. 1). Malaria transmission in the area is low but seasonal, and peaks during the rainy season from March to August. The Osudoku Community Health Centre is the main health care facility in the area. In November 2013, 184 children (aged 2–15 years), 154 male and non-pregnant female adults (aged 16–65 years) and 125 pregnant women (aged 18–45 years) were recruited and followed-up for a period of 10 months or until delivery (for pregnant women).

### Sampling and morbidity surveillance

At enrolment, 5 ml of EDTA anti-coagulated venous blood was collected from each participant at baseline (November 2013). A small aliquot (50 µl) was used to prepare thick and thin blood films for microscopy. Plasma was separated from the rest of the whole blood by centrifugation at 450×*g* for 10 min; plasma was aliquoted and stored at − 20 °C until use. During follow-up, cross sectional sampling was done in February 2014 (Follow-up 1, dry season), May 2014 (Follow-up 2, rainy season) and September 2014 (Follow-up 3, end of rainy season) in which finger prick blood (300 µl) for detection of asymptomatic parasitaemia was taken during follow-up visits. Clinical malaria was defined as microscopy slide positive for any parasitaemia plus fever (axillary temperature > 37.5 °C), together with at least one other symptom such as malaise, vomiting or diarrhoea. Spotted filter papers were stored at − 20 °C in zip lock bags with silica gel desiccant for molecular analysis. For gametocyte detection, 50 μl whole blood samples were preserved in 250 μl RNAlater^®^ solution (Ambion, Life Technologies) and stored at − 20 °C until use. Weekly active surveillance was conducted by home visit, with a standardized questionnaire to capture clinical information for all participants. Participants who became ill during follow-up periods were referred to the community health centre for treatment per the Ghana Health Service guidelines. In between visits, participants were advised to go on their own to the health centre when they felt ill.

### Laboratory procedures

Blood haemoglobin levels were measured using the Hemocue-Hb 201 (Angelholm, Sweden). The ABO blood type was determined using a commercial anti-sera blood grouping kit (Biotec Laboratories Limited, UK). All participants were screened for their sickle cell status using the sodium metabisulphite method.

### Parasitaemia by microscopy

Thick and thin blood smears were prepared and stained with 10% Giemsa for detection of both asexual stage and sexual stage parasites by microscopy under oil immersion (100× magnification). Parasite densities were assessed by counting 200 leucocytes, and converted to number of parasites per μl of blood by assuming a standard leucocyte count of 8000/μl. A slide was considered negative if no parasites were seen after examination of at least 200 fields.

### Submicroscopic asexual parasite detection

DNA was extracted from blood samples blotted on filter paper by the Chelex method as previously described [23]. *Plasmodium* species were determined by nested polymerase chain reaction (PCR) methods as previously described [24], with modifications. Briefly, *Plasmodium* genus were detected using the outer genus-specific primers (rPLU 5 & 6) targeting sequences of their small subunit ribosomal (ssrRNA) genes. The initial outer reaction contained 4 mM of MgCl_2_, 200 µM DNTPs, 0.0625 μM of each primer and one unit of Taq DNA polymerase (Sigma-Aldrich, USA). The PCR cycling conditions for the primary reaction were an initial denaturation at 94 °C for 15 min, denaturation for 1 min at 94 °C and annealing at 58 °C for 2 min (all for 30 cycles). Extension was at 72 °C for 2 min, a final annealing at 58 °C for 2 min and final extension at 72 °C for 10 min. The inner PCR reaction was used for detection of all *Plasmodium* species as described in [25]. The cycling conditions and number of PCR cycles was as above except for annealing that was set at 55 °C for 2 min. All PCR reactions were performed using a GeneAmp PCR System 2700 (Applied Biosystems Incorporated, US).

### Gametocyte detection

RNA extraction was done using RNeasy^®^ plus mini kit (Qiagen, USA or Germany) according to the manufacturer’s protocol for selected RNA-preserved samples that were asexual parasite positive by microscopy and/or PCR. Gametocyte detection was done by *Pf*s25 real time quantitative PCR [26]. Even though the *Pf*s25 mRNA is transcribed only by female gametocytes, gametocyte sex ratios are female biased and the marker has therefore been used as a sensitive marker for circulating matured gametocytes [27, 28]. Briefly, RT-PCR targeting *Pf*s25 transcripts was performed with the primer sequences *pfs*25_fw 5′-GAA ATC CCG TTT CAT ACG CTT G-3′, pfs25_rev 5′-AGT TTT AAC AGG ATT GCT TGT ATC TAA.-3′ and *Pf*s25_probe HEX-TGT AAG AAT GTA ACT TGT GGT AAC GGT-BHQ1. The RT-PCR reaction mixture contained an enzyme mixed reagent, a master mix solution*, Pfs*25-specific forward and reverse primers at concentrations 800 nM, *Pf*s25_probe at 200 nM, and 2 µl of RNA template. The RNA template for the reaction included 3D7 gametocyte-positive standard RNA generated from culture with concentrations starting from 10,000 to 0.005 gametocytes/μl and a parasite-negative sample, as well as a no template control. The reaction plate was loaded into an ABI 7300 real-time PCR instrument (Applied Biosystems Incorporated, USA). The cut-off threshold (C_T_) for positivity was set to ≤ 37.0 C_T_ value by using a purified gametocyte standard from a 3D7 parasite culture.

### Multiplicity of infection by *msp2* genotyping

The *P. falciparum* positive samples were selected for the detection of multiple parasite clones by nested PCR using msp2 family-specific primers [23]. Briefly, the initial outer reaction was performed using oligonucleotide primers with conserved sequences to msp2 and inner PCR primers were used to amplify FC27 and 3D7 family variants. Purified DNA from the 3D7 and Dd2 *P. falciparum* laboratory strains were used as positive controls and a negative control consisting of only working reagents was used. The amplified products were run on a 2% ethidium bromide-stained agarose gel and visualized using an ultraviolet transilluminator. Multiplicity of infection was calculated by dividing the number of *msp2* bands obtained from the gel by the total number of positive samples run. Samples with single bands were considered as monoclonal infections while samples with multiple bands were termed multiclonal infections.

### Anti-*Pf*s230 gametocyte antibody measurement

Recombinant *Pf*s230D1M (GenBankTM accession number XP_001349600.1) was characterized from *Pf*s230 domain 1 (*Pf*s230D1H) and non-structured domain 1–2 (*Pf*s230D1-2). The recombinant protein was refolded by removing the His_6_ fusion tag and expressed in *Pichia pastoris* using good manufacturing practice conditions and purified by RP-HPLC as previously described [29].

*Pf*s230-specific antibody levels were measured by indirect ELISA. Briefly, 96-well microtitre (Maxisorp, Nunc, Denmark) plates were coated with 1 µg/ml of recombinant *Pfs*230D1M in carbonate buffer (pH = 9.2) and incubated overnight at 4 °C. Plates were washed 4 times with washing buffer (PBS with 0.05% Tween-20) and blocked in 3% skimmed milk powder in PBS, 0.05% Tween-20 for 1 h. Plasma samples were diluted 1:300 in dilution buffer (1% non-fat dry milk in PBS with 0.5% Tween 20) and incubated for 2 h at room temperature. A positive control of pooled hyper immune plasma was titrated as a standard and plasma from malaria-naïve US volunteers as negative controls were included on each plate as internal controls. Bound antibodies were detected with 1:3000 diluted peroxidase conjugated goat anti-human IgG (H+L) (ThermoFisher Scientific, USA) in dilution buffer and incubated for 1 h at room temperature. After washing 4 times, plates were developed with tetramethylbenzidine substrate (TMB plus 2, KEM-EN-TEC Diagnostics, Denmark) for 15 min in the dark and the reaction stopped with 0.2 N H_2_SO_4_. Absorbance was read at 450 nM on an ELISA plate reader (Biotek Instruments, USA).

### Statistical analysis

Study data were entered into a Microsoft Access database and exported into Microsoft Excel for statistical analysis. The difference in proportions of covariates including age, sickle cell, bed net use, haemoglobin levels and blood group among the groups were compared using Pearson’s Chi squared test and ANOVA. The association between asexual parasitaemia and/or gametocytes and multiple infections were tested independently using multiple logistic-regression analyses. Gametocyte prevalence in relation to the proportion of *msp2*-3D7 and *msp2*-FC27 clones was assessed using quasi-Poisson regression. For the study period, samples that were *P. falciparum* positive at least one-time point throughout the entire study period were included in data analysis. The ELISA OD values were transformed into arbitrary antibody units (AU) using the Auditable Data Analysis and Management System for ELISA (ADAMSEL FPL version b040, Ed Remarque^®^, Netherlands). Pairwise comparison amongst median antibody levels across groups and within groups at various time points was done using Bonferroni comparison test following Kruskal–Wallis analysis. The mixture model [30, 31] was used to define a cut-off value above which samples were categorized as antibody-positive. The distribution of normalized OD values was fitted as the sum of two Gaussian distributions which yielded a narrow distribution of seronegatives and a broader distribution of seropositives. The mean OD of the Gaussian corresponding to the seronegative population, plus three standard deviations, was used as cut-off for seropositivity. All graphics were made using the Graph pad prism software (version 5, San Diego, USA) and all statistical analysis were performed with the R statistical package (R version 3.3.2, 2016) For all analysis, the P-value was set at 0.05% level of significance.

## Results

### Demographic and clinical characteristics of the study population at baseline

A total of 463 samples from the three study groups were analysed at baseline, consisting of 184 children, 154 non-pregnant adults and 125 pregnant women (Table 1). There was similar low proportions of sickle cell trait among the study groups but haemoglobin levels were highest in the adults group and lowest among pregnant women (*P < 0.001). Bed net usage was highest among pregnant women compared to children and adults (P < 0.001, Table 1).

**Table 1:** Demographic and clinical characteristics of study groups

Variables n=	Children 184	Adults 154	Pregnant women 125	P value
Age, median (IQR)	8 (5–11)	30 (23–38)	24 (20–28)	–
Gender				
Female	95 (51.6%)	117 (76.0%)	125 (100%)	–
Male	89 (48.4)	37 (24.0%)	–	
Sickle cell trait				
Positive	11 (6.0%)	5 (3.3%)	2 (1.8%)	0.18
Negative	173 (94.0%)	145 (96.7%)	110 (98.2%	
Blood group				
A	33 (17.9%)	28 (18.2%)	26 (23.0%)	0.492
AB	14 (7.6%)	6 (3.9%)	6 (5.3%)	
B	51 (27.7%)	49 (31.8%)	38 (33.6%)	
O	86 (46.7%)	71 (46.1%)	43 (38.1%)	
Haemoglobin levels mean, (SD)	11.1 (1.3)	11.9 (1.6)	9.9 (1.5)	< 0.001*
Bed net usage				
Yes	103 (56.0%)	88 (59.1%)	98 (78.4%)	0.001
No	81 (44.0%)	61 (40.9%)	27 (21.6%)	
Asexual parasite density by microscopy	1180 (80–17,680)	1120 (80–20,412)	1720 (320–8480)	0.61**
Gametocyte density by RT-PCR	1.43 (0.01–1267)	0.04 (0.02–0.08)	0.54 (0.01–6.04)	0.47**

P values were calculated by Pearson’s Chi squared test, and * ANOVA P value ≤ 0.05 is significant. *IQR* inter quartile range. ** P value was calculated by Kruskal–Wallis test. Parasite density/µl of blood (range)

### Distribution of *Plasmodium* species and prevalence of asexual stage parasites

**Fig. 2:**
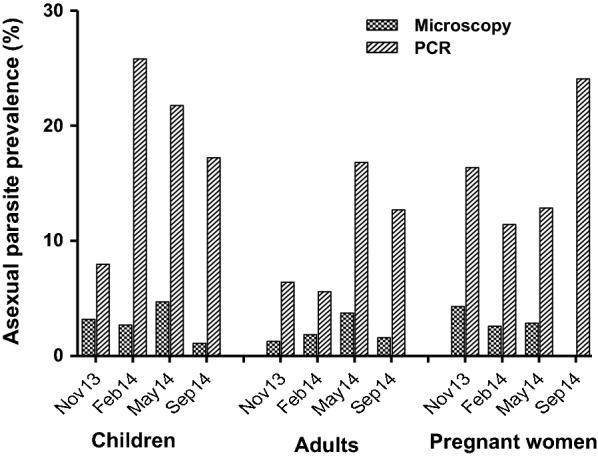
Asexual parasite prevalence among the study groups at the various time points. Enrolment (November 2013) and follow-up time points. Follow-up 1 (February 2014), Follow-up 2 (May 2014) and Follow-up 3 (September 2014) among the groups: Children, Adults and Pregnant women detected by both microscopy and PCR

The pattern of asexual parasite prevalence between study groups was similar for microscopy and PCR data, and prevalence estimates by PCR were higher as expected (Fig. 2). The PCR data is therefore subsequently presented and used in all analysis since all samples were analysed by both methods. Generally, the parasite distribution in the study community was predominantly *P. falciparum* (96%); *Plasmodium malariae* and *Plasmodium ovale* species were less than 5% and were detected either as single infections or co-infections with *P. falciparum.* The prevalence of asexual *P. falciparum* parasitaemia among the adults at baseline (November 2013) was generally lower (6.4%) compared to that in children (8.0%) and pregnant women (16.4%), (Fig. 2, Additional file 1). Asexual parasite prevalence varied during the follow-up periods in which children had higher parasite prevalence, especially during the beginning of the rainy season compared to adults Fig. 2, Additional file 1). Also, pregnant women had relatively higher parasite prevalence compared to adults throughout the survey periods (Fig. 2). The median asexual parasite densities were higher in children and pregnant women compared to adults though not significant (P = 0.61, Table 1).

### Prevalence of submicroscopic gametocytes in the study population

Due to the low prevalence of gametocytes, the gametocyte levels over the entire study period was used for the analysis. Focusing on the PCR positive data, gametocyte prevalence among children was 39.5% (15/38) by *Pfs*25-based RT-PCR. The adult and pregnant women’s group had gametocyte prevalence of (17.4%, n = 4/23; 29.7%, n = 11/37 respectively). Based on the RT-PCR data, children had high median gametocyte densities compared to pregnant women and adults in the study population (P = 0.47, Table 1).

### Prevalence and multiplicity of *Plasmodium falciparum* infections and association with gametocyte carriage

During the entire study period, asexual parasite-positive samples detected by PCR were used for the detection of multiplicity of infection (MOI). Most of the *P. falciparum* positive samples (87.1%) had multiple alleles (≥ 2 PCR bands) and few infections were single alleles (12.9%).

From the study, overall MOI amongst children and pregnant women were found to be higher compared with adults (P = 0.006, Table 2). Pairwise comparisons indicated a significant difference in the mean number of clones between pregnant women and adults (P = 0.009) and children (P = 0.025). When the association between family-specific type of clones (3D7 and/or FC27) and gametocyte prevalence was examined, it was observed that an infection with both 3D7 and FC27 family types significantly increased the odds of gametocyte carriage (OR = 5.92, 95% CI 1.56–22.54) compared to only FC27 or 3D7 allelic types (Table 3).

**Table 2:** The distribution parasite density and mean number of clones among the groups

Study groups	Geometric mean parasite density	Number of clones of clones/sample
Children	1510 (634–3597)	3.13 ± 1.11
Adults	3959 (301–52,041.86)	2.75 ± 0.62
Pregnant women	2062 (865.97–4907.68)	3.90 ± 1.14
*P*-values	0.62	0.006

P-values were calculated from one-way ANOVA, Mean number of clone’s ± Standard deviation

**Table 3:** Association between *P. falciparum* msp2 allelic family type and gametocyte carriage

Infecting clone type	Gametocytes (n) Positive	Negative	OR	95%CI	P-value
3D7	24	46	1		
FC27	24	22	7.0	0.34–144.08	*0.207*
3D7 + FC27	22	26	5.92	1.56–22.54	*0.009*

Odds ratio (OR), 95% confidence interval (CI), calculated from logistic regression analysis. n: is the sample size for gametocytes

*P* value ≤ 0.05 in italic

### Anti-Pfs230 median antibody levels and seroprevalence among the three study groups

**Fig. 3:**
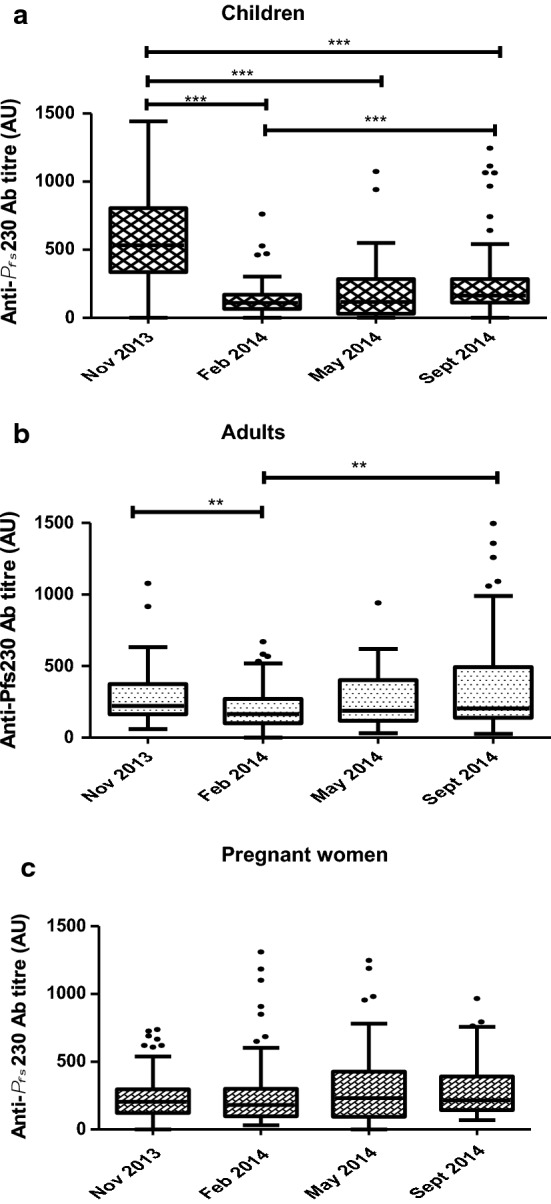
Anti-*Pfs*230 median antibody levels among the different study populations. **a** Antibody levels for Children’s group,** b** is antibody levels for Adults;** c** antibody levels for pregnant women. P value ≤ 0.05 is significant by Kruskal–Wallis test and Bonferroni test. Error bars indicate the upper and lower limits of the 95% confidence interval around the proportion

Antibody levels for each group varied at different time points, however that of adults remained relatively stable at the different follow-up periods except for follow-up 3 in September 2014 (Fig. 3; P < 0.05). The effect of age on anti-*Pf*s230 antibody levels was assessed in children and the older (adults and pregnant women) individuals separately. Age was significantly associated with increased anti-*Pfs*230 antibody levels in both children (β = 0.80, 95% CI 0.42–1.19, P < 0.001) and the older individuals (β = 1.66, 95% CI 0.09–3.24, P = 0.039).

**Fig. 4:**
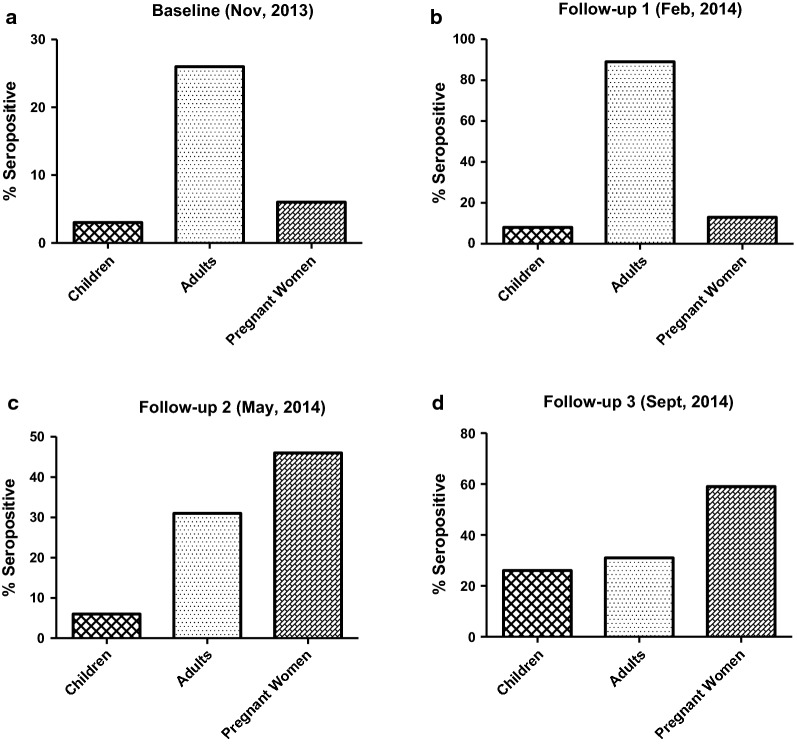
IgG Seropositives among the three different groups. Antibody seropositive responses for *Pf*s230 was done using the mixture model, which shows a bimodal distribution into seropositive and seronegative populations. Cut-off for seropositivity was defined as the mean of the seronegative individuals from the Gaussian distribution plus three times the standard deviation.** a** Baseline, November 2013; Children (5/159), Adults (35/133), Pregnant women (8/123);** b** follow-up 1, February 2014: Children (14/175), Adults (81/91), Pregnant women (10/79);** c** follow-up 2, May 2014; Children (5/85), Adults (11/34), Pregnant women (27/58);** d** follow-up 3, September 2014 Children (43/164), Adults (33/108), Pregnant women (32/54)

The seroprevalence data was generated using seropositivity cut-off values estimated by a mixture model. All data that were available at the various time points were used for this estimates. For the adults group, there was a high baseline seroprevalence of anti-*Pfs*230 antibodies (26%). However, the seroprevalence of antibodies were relatively lower in children (3%) and in pregnant women (6.6%) (Fig. 4a). During the entire follow-up period, anti-*Pfs*230 antibody seroprevalence remained higher in adults and pregnant women compared to children.

**Fig. 5:**
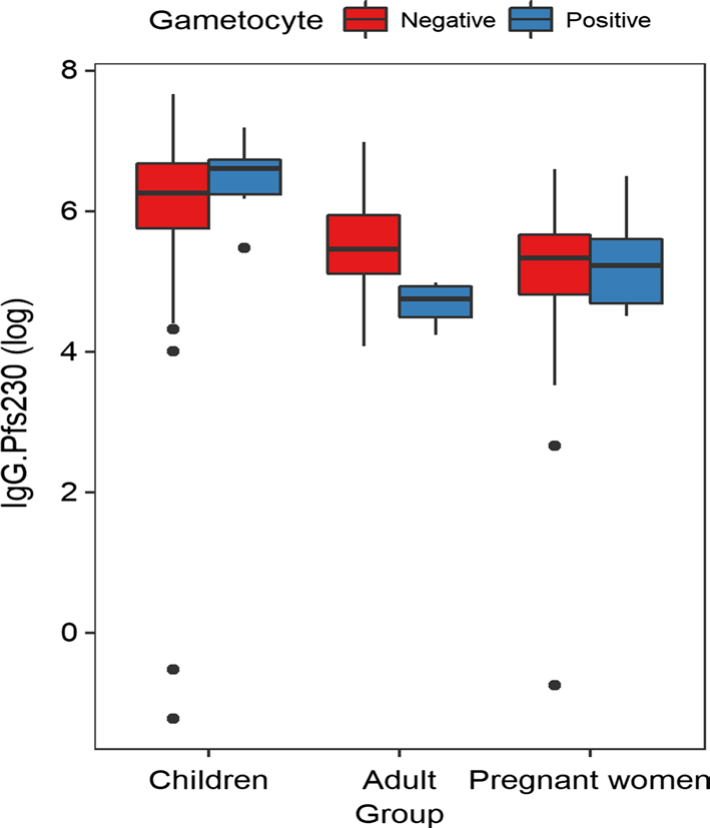
Anti-*Pf*s230 levels and gametocyte positive and negative individuals among the groups. Association was determined by linear regression

The association between asexual parasite prevalence and antibody responses to *Pf*s230 was not significant. When *Pf*s230 antibody responses were compared between gametocyte negative and positive individuals, the levels were significantly different in the adults (β = − 0.57, 95% CI = − 0.81,− 0.34, P < 0.001) and pregnant women (β = − 0.81, 95% CI − 1.05,− 0.57, P < 0.001) but not in the children (Fig. 5).

## Discussion

The gametocyte stage of *P. falciparum* is essential for malaria transmission. Since studies have shown that even low levels of gametocytes may lead to effective transmission of malaria [10, 12]), it is important to identify submicroscopic gametocyte reservoirs in low transmission areas to help in effective control measures. A number of factors have been implicated to influence gametocyte carriage, and these include the infecting parasite load and simultaneous presence of multiple parasite clones in a single infected individual among others [32, 33].

The current study investigated the association of asexual parasite density and multiple parasite clone infections with gametocyte carriage in three different demographic groups in a low malaria transmission area. Using PCR, a relatively high prevalence of asymptomatic infections and gametocytaemia were detected among children and pregnant women compared to adults. The immune systems of children and pregnant women have been shown to be less robust compared to that of non-pregnant adults [16, 34] and this may explain the higher prevalence of parasites in the two former groups compared to adults. Furthermore, the difference in the asexual parasite prevalence could also be due to seasonal variations as a result of different transmission intensities at the various time points [30]. Lastly, in pregnant women, parasite variants that sequester in the placenta evade the host immune system and may reach very high parasite densities which cannot be detected in peripheral blood, and these may persist and develop into gametocytes [35, 36]. In this study, only submicroscopic gametocyte infections were detected in pregnant women, this could be due to low levels of circulating gametocytes, hence none was detected by microscopy. In general, majority of the gametocytes detected were submicroscopic in this low transmission area, in agreement with findings from other parts of southern and coastal Ghana [20, 37].

The overall MOI observed in this study was high (3.1) for such low transmission area. This finding was strange and unexplainable, since studies have shown higher MOI in high transmission areas due to more parasite diversity and associated with transmission intensity, compared to low transmission areas with limited parasite diversity [38, 39]. This finding though in a low transmission area, was similar to what was observed from independent studies conducted in a high transmission area in the Ashanti region of Ghana, in which multiplicity of infection ranged between 1.96 and 3.39 among asymptomatic populations [18, 40].

There was a high proportion of multiclonal infections among the children and pregnant women groups in this study compared to adults. Pairwise comparison revealed that pregnant women had higher mean number of clones than children and adults. This is an indication that they carry a greater diversity of parasites, possibly reflecting infection with both placenta-specific and non-specific parasites, especially in primigravids [41–43]. Interestingly, the number of clones was observed to increase in individuals carrying gametocytes as compared to those who did not carry gametocytes. These multiclonal infections may have persisted in these individuals and enhanced gametocyte carriage, to cause an increased chance of some of the parasite variants evading the host immune system and committing to gametocyte formation [17]. Other studies have also shown MOI in children to be higher compared to that in adults [32, 44]. However in studies conducted in some coastal parts of Ghana involving children, MOI was not associated with gametocyte prevalence [20, 37].

The 3D7 allelic types predominated in all the three study groups as compared to the FC27 allelic types, indicating its dominance in this geographical area. A number of studies conducted in Ghana and Tanzania have identified the 3D7 allelic parasite type to be dominant among the parasite populations [18, 40, 45, 46]. Different *P. falciparum* parasite variants may influence gametocyte carriage differently, as was observed in this study where individuals carrying both 3D7 and FC27 allelic types of parasite had a higher risk of harbouring gametocytes compared to single 3D7 or FC27 allelic types. This findings may suggest the possibility of limited immune responses against these parasite variants due to antigen-specific immunosuppression or immune tolerance, hence their ability to persist for longer periods and commit to gametocyte formation [47]. However, in other studies, the family-specific FC27 allelic type parasites have been shown to be associated with high parasite densities as well as involved in disease severity, suggesting they may be more pathogenic [46, 48]. This underscores the need to investigate the potential role other family allelic type of parasites may play in gametocyte carriage in different study populations and transmission areas, since its effect may impact on transmission.

This study also investigated the seroprevalence of antibody responses to *Pf*s230 antigen among different study groups and found the adult study group with the highest anti-*Pf*s230 seroprevalence as compared to children and pregnant women at baseline. Difference between adults and children may probably reflect repeated exposure in adults, as has been observed in other studies [21, 49, 50]. The lower seroprevalence of antibodies at baseline in the pregnant women’s group may perhaps be due to fewer submicroscopic gametocytes detected. However, during the follow-up period, seroprevalence of antibodies varied among the groups at different transmission seasons. This could be due to the half-life of sexual stage-specific immunity that has been shown to be in a range of 1–3 months [21, 51, 52]. An indication of current exposure to gametocytes during the various transmission seasons, as levels of sexual stage-specific immune responses have been demonstrated in other studies to depend on recent exposure, rather than cumulative exposure [21, 53]. For instance, the decrease in anti-*Pf*s230 seroprevalence observed in the adults group between February and May could be due to reduced levels of circulating submicroscopic gametocytes over the period. Interestingly, antibody responses to *Pf*s230 were significantly lower in adults and pregnant women who had gametocytes compared to children, meaning the responses in children might have been boosted by recent exposure to *Pf*s230 antigens since children were found to harbour more gametocytes. For the adults, the lower gametocyte densities may indicate some form of anti-gametocyte immunity as proposed by other studies [7, 54]. However, evidence to this is divergent, since sexual stage immunity has been shown to induce transmission blocking responses in the mosquito, and may not offer anti-gametocyte immunity directly to the host [55]. In this study, age was significantly associated with increased *Pf*s230 levels among the groups, suggesting the length of gametocyte exposure among the groups. This age-dependent association of sexual stage antibody levels have been observed in some studies [21, 53, 56], but not in others [50, 52].

## Conclusions

Multiplicity of infection was higher in gametocyte carriers than non-gametocyte carriers, suggesting an association between simultaneous presence of multiple parasite clones and gametocyte carriage. This needs to be further evaluated in other transmission areas since it may impact on transmission. Antibody responses to *Pfs*230 in children was associated with gametocyte carriage, an indication of it being a marker for current gametocyte exposure.

## Additional file

**Additional file 1.** Asexual parasite prevalence data among the groups at various time points by Microscopy and PCR. Baseline, November 2013; F1-3 are follow-ups 1-3 (February 2014, May 2014 and September 2014) respectively.

## Abbreviations

ACT: artemisinin-based combination therapy
C_T_: cut-off threshold
DNA: deoxyribonucleic acid
ELISA: enzyme linked immunosorbent assay
MOI: multiplicity of infection
MSP: merozoite surface protein
OD: optical density
RP-HPLC: reverse-phase high performance liquid chromatography
RNA: ribonucleic acid
RT-PCR: real time reverse transcription polymerase chain reaction
