# Supplementary material for: The prevalence of submicroscopic Plasmodium falciparum gametocyte carriage and multiplicity of infection in children, pregnant women and adults in a low malaria transmission area in Southern Ghana
Source: Malar J. 2018 Sep 17;17:331. doi: 10.1186/s12936-018-2479-y (PMC6142636; doi:10.1186/s12936-018-2479-y)
Supplement: Supplementary file 1 — Additional file 1. Asexual parasite prevalence data among the groups at various time points by Microscopy and PCR. Baseline, November 2013; F1-3 are follow-ups 1-3 (February 2014, May 2014 and September 2014) respectively. [file 12936_2018_2479_MOESM1_ESM.docx]

**Additional File 1: Asexual parasite prevalence data for the groups at the various time points by Microscopy and PCR**

| **Group** | **Microscopy (%)** | **Submicroscopic (%)** | **Overall (%)** |
| --- | --- | --- | --- |
| **Children** | | | |
| Baseline (n=184) | 3.26 | 4.89 | 8.15 |
| F1 (n=182) | 2.75 | 24.18 | 26.92 |
| F2 (n=170) | 4.71 | 17.06 | 21.76 |
| F3 (n=180) | 1.11 | 17.22 | 18.33 |
| **Adults** | | | |
| Baseline (n=154) | 1.3 | 5.19 | 6.49 |
| F1 (n=141) | 2.13 | 4.26 | 6.38 |
| F2 (n=107) | 3.74 | 13.08 | 16.82 |
| F3 (n=126) | 1.59 | 12.7 | 14.29 |
| **Pregnant Women** | | | |
| Baseline (n=126) | 4 | 11.2 | 15.2 |
| F1 (n=77) | 2.6 | 7.79 | 10.39 |
| F2 (n=70) | 2.86 | 10 | 12.86 |
| F3 (n=54) | 0 | 24.07 | 24.07 |

**Baseline, November 2013; F1-3 are follow-ups 1-3 (February 2014, May 2014 and September 2014) respectively**
